# Estimation of the incidence of hospitalisation for non-invasive pneumococcal pneumonia in the Norwegian population aged 50 years and older

**DOI:** 10.1017/S0950268822000607

**Published:** 2022-04-04

**Authors:** Trude Marie Lyngstad, Anja Bråthen Kristoffersen, Brita Askeland Winje, Anneke Steens

**Affiliations:** 1 Norwegian Institute of Public Health, Oslo, Norway; 2 European Programme for Intervention Epidemiology Training (EPIET), European Centre for Disease Prevention and Control (ECDC), Stockholm, Sweden

**Keywords:** Non-invasive pneumococcal pneumonia, Pneumococcal pneumonia, Streptococcus pneumonia, incidence, duration of hospitalisation

## Abstract

The purpose of this study was to estimate simple measures of the burden of non-invasive pneumococcal pneumonia (PnPn) hospitalisations in those aged 50 years and older (50+) in Norway. We conducted a retrospective register-based study and used discharge codes from the Norwegian Patient Register (NPR). We identified episodes of non-invasive PnPn in 2015 to 2016 and predicted its incidence from 2015 to 2019 based on the trend found in notified invasive pneumococcal disease cases. Overall, we identified 45–46 hospital episodes per 100 000 population of non-invasive PnPn in 2015 and 2016, each episode taking 6–8 days, and with increasing incidence with higher age. Among all identified PnPn episodes, 3 out of 4 were classified as non-invasive. We predicted that the monthly number of non-invasive PnPn episodes ranges from 39 [95% confidence interval (CI) 24–55] in August to 97 (95% CI 74–134) in December. No annual trend was identified. This study indicates that the burden of non-invasive PnPn hospitalisation has a substantial impact on the health and health care use of the 50+ population in Norway, despite the childhood immunisation programme. Many hospitalisations may be prevented through vaccination.

## Introduction

The bacterium *Streptococcus pneumoniae*, commonly called the pneumococcus, is a normal inhabitant of the human upper respiratory tract, *i.e.*, colonises the nasopharyngeal tract, especially in children. *S. pneumoniae* can cause infections in many parts of the body, and may cause pneumonia, otitis, sinusitis, meningitis and sepsis.

*S. pneumoniae* is the most common aetiology in community-acquired pneumonia (CAP) and is reported to be responsible for 20% of all adult CAP-cases in Europe [1], despite its challenges in diagnosis [2]. The observed prevalence varied significantly between regions in Europe [1]. In a recent study from the UK, 37% of all CAP were caused by *S. pneumoniae*, and these authors also reported that the annual pneumococcal CAP had increased from 2013 to 2018 [3].

Invasive pneumococcal disease (IPD), defined as isolation of *S. pneumoniae* from a normally sterile site, is a notifiable disease in many countries including Norway. According to the Surveillance System for Communicable Diseases (MSIS) the annual number of cases ranged from 522 to 599 in the years before the coronavirus disease 2019 (COVID-19) pandemic (2015–2019) [4]. The annual number of notified IPD dropped to 295 during the COVID-19 pandemic year 2020, of which 241 (82%) occurred in the 50+ population. Non-invasive disease such as non-invasive pneumococcal pneumonia (PnPn) is not notifiable. Therefore, we do not have an overview of its epidemiology, although hospitalised cases of non-invasive PnPn can be found in the Norwegian Patient Register (NPR) using discharge diagnoses. Non-invasive PnPn is less severe than IPD, however the incidence is much higher and thereby it contributes substantially to the burden of pneumococcal disease.

In a meta-analysis, the estimated proportion of all PnPn that was invasive was 25% [95% confidence interval (CI) 21–29] [5]. In agreement with these findings, a more recent study from the Netherlands (among 409 patients) found that 30% of the PnPn episodes were invasive and 70% non-invasive [6]. Both, young children, elderly and persons with weakened immune systems are at high risk of acquiring pneumococcal disease [7, 8].

The currently available 13-valent pneumococcal conjugate vaccine (PCV13) and the 23-valent polysaccharide vaccine (PPV23) are both safe and efficacious in preventing invasive and non-invasive pneumococcal disease [9]. Vaccination against pneumococci with PCV13 is included in the national childhood immunisation programme (NIP) in Norway since 2011. The PPV23 is recommended for older adults (65+) and persons belonging to risk groups *i.e.* persons with underlying diseases [10]. PCV13 in combination with PPV23 is only recommended for a few high-risk groups. However, as of today, there is no national immunisation programme for pneumococcal vaccination for older adults (50+) in Norway, therefore costs are not reimbursed. Uptake has been low (around 15%) and differences related to social, economic and place of residence (geographical factors) may occur [8]. Because of the increasing proportion of 50+ population in Norway, likely resulting in an increasing demand for health care in the next few decades, preventing infectious diseases through immunisation will be essential [11].

The Norwegian Institute of Public Health (NIPH) is participating in the project on Vaccines and Infectious Diseases in the Ageing Population (VITAL) [12]. The project aims to quantify the impact of vaccination strategies for protecting the ageing population against infectious diseases. The VITAL project has published a review of gaps of data and potential data sources for burden of disease. However, PnPn specific data is lacking for Norway as well as for most European countries [13].

In order to inform and improve adult-vaccine policy for pneumococcal vaccination in Norway we need, in addition to the burden of IPD, to better understand the burden caused by non-invasive PnPn in the 50+ population. We therefore aimed to estimate simple measures of the burden of non-invasive PnPn hospitalisations in the 50+ population. Furthermore, we aimed to predict the number of hospitalised non-invasive PnPn episodes in 2015–2019 using hospital discharge data for 2015–2016 and to extrapolate to 2019 based on the trends found in IPD.

## Methods

### Study design, data and definitions

**Fig. 1.**
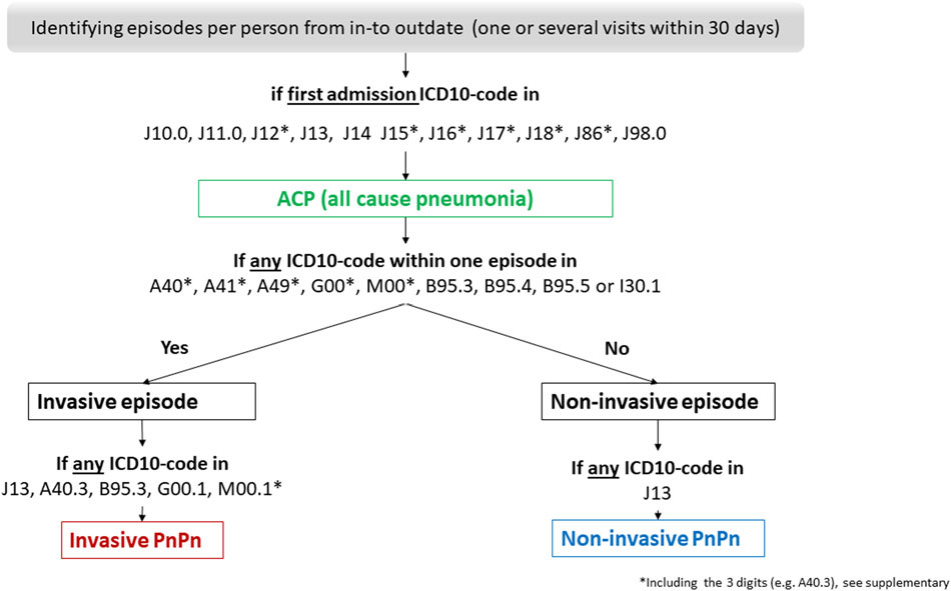
Flowchart; generating variables from hospital discharge data on all cause pneumonia (ACP), invasive and non-invasive pneumococcal pneumonia (PnPn).

This study is a retrospective register-based study. The study population is the Norwegian population aged 50+ in 2015–2019 (34%–36% of the total population depending on the year, Statistics Norway). We used hospital discharge data from NPR. A selection was made from NPR that included all patients 50+ (aged 50 years and older) in 2015–2016 with a discharge code according to ICD-10 codes that might be pneumococcal diseases (Fig. 1). Age, dates and discharge diagnoses for all their episodes from all hospitals in Norway in the study period were included in the dataset. These data were obtained as part of previous European studies that included Norwegian data [14, 15]. Ethical clearances for the usage of the hospital discharge data in this study were obtained from the Regional Committees for medical and health research ethics (REK, No 28638 and 28635). Data on the number of IPD cases notified to the MSIS, stratified by month, year (2015–2019) and age group were retrieved from the web [4], see also Table S1. The population data were obtained from Statistics Norway, open source. As the study period was pre-COVID-19, the IPD and non-invasive PnPn incidence was not affected by the COVID-19 infection prevention and control measures.

All-cause pneumonia (ACP) was defined as a first-listed discharge diagnosis of pneumonia according to the ICD10-codes J10.0, J11.0, J12*, J13, J14, J15*, J16*, J17*, J18*, J86*, J98.0 (*including the 3 digits). The invasive disease was defined according to any of the following ICD-10 codes A40*, A41*, A49*, G00*, M00*, B95.3, B95.4, B95.5 or I30.1. We defined episodes per person as one or several admissions for ACP within the same 30 days. If there were any ICD-10 code that defined an invasive disease, the disease status of the episode was set to invasive, otherwise diseases status was set to non-invasive. Non-invasive PnPn was defined according to ICD-10 code J13 but without having any ICD-10 code that reflects invasive disease within the episodes (*i.e.* within 30 days). Note that a PnPn case will often be coded as J13 based on a combination of symptoms and radiological findings with supporting laboratory evidence of pneumococcal aetiology. Invasive PnPn was defined according to the invasive ICD-10 codes, and/or at least one of the following codes J13, A40.3, G00.1 and M00.1*, B95.3 (*including the 3 digits, Fig. 1).

Cases of IPD and hospital episodes of non-invasive PnPn were aggregated by year, month and age group. Descriptive statistics for the number of episodes, duration of hospital stay and readmissions were calculated per age group according to standard procedures. Incidences were calculated using the Norwegian population size as the denominator. To obtain denominator data per month, we added growth as a linear change per month based on the size of the population at January of the next year, to account for changes in the population size.

### Modelling approach

We aimed to predict the monthly number of non-invasive PnPn hospital episodes for the period from 2015 up to 2019. Hospital data on non-invasive PnPn were available for two years (2015–2016), which is a period too short to use for predicting the following years. However, the trend has been found to be similar for IPD and non-invasive PnPn [3, 6, 16]. We therefore explored this similarity and then predicted non-invasive PnPn trends based on IPD trends.

To predict the IPD trend, we updated a previous published quasi-Poisson model [17] using IPD data on those aged 50+ (IPD model). Because of changes in the childhood immunisation programme in 2011 and subsequent redistribution of the serotypes [18], we choose to use data on IPD counts from 2015 onwards. Explanatory variables that were tested in the regression model were *year* and *month* (*year* as a continuous variable and *month* as a factor). Akaike information criterion (AIC) was used to choose the best model. To account for changes in the population size we used the estimated Norwegian population at a given month (*pop*).

We modelled associations between monthly episodes of non-invasive PnPn and IPD, comparing both linear and log-linear regression models (non-invasive PnPn model).

Based on the *IPD model* we predicted the number of episodes with 95% CIs for each month from 2015 to 2019. Then these predicted IPD episodes (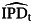) were further used as a time-dependent explanatory variable in the *non-invasive PnPn model* to predict the number of non-invasive PnPn episodes (the dependent variable). To predict the lower/upper bounds of the 95% CI for non-invasive PnPn, we used the corresponding lower/upper bounds of the 95% CI from the IPD model in the non-invasive PnPn model. We checked the data on IPD and non-invasive episodes for extreme observations and performed a sensitivity analysis by running the modelling approach without these.

The statistical analyses were conducted in R version 4.0.2 (The R Foundation for Statistical Computing).

## Results

We identified 44 876 episodes among 37 633 individuals that were hospitalised with ACP as the first listed discharge diagnosis (Fig. 1). Among all hospitalised ACP episodes, 5% were identified as being caused by pneumococci, i.e., PnPn (2015; 1106/22 543 and 2016; 1107/22 333 Table 1). Among all identified PnPn episodes a total of 73% (812 of 1106 PnPn episodes in 2015) and 74% (821 of 1107 PnPn episodes in 2016) were identified as non-invasive PnPn.

**Table 1.** The number of episodes of all-cause pneumonia, non-invasive pneumococcal pneumonia, invasive pneumococcal pneumonia and invasive pneumococcal disease in the population aged 50 years and older in Norway (2015–2016), based on ICD-10 discharge codes defined per person within the same 30 days of hospitalisation

Disease	2015 Episodes	2015 per 100 000	2016 Episodes	2016 per 100 000
All-cause pneumonia^a^	22 543	1271	22 333	1235
Non-invasive pneumococcal pneumonia^a^	812	46	821	45
Invasive pneumococcal pneumonia^a^	294	17	286	16
Invasive pneumococcal disease (MSIS)^b^	431	24	488	27

a Data source: Norwegian Patient Register (NPR).

b Data source: Surveillance System for Communicable Diseases (MSIS).

We identified 45–46 hospital episodes per 100 000 population (*n* = 812 and 821) of non-invasive PnPn in 2015 and 2016 for the 50+ (Table 1). The number of episodes of non-invasive PnPn per 100 000 increased by age, and ranged from 15 and 16 per 100 000 among those aged 50–59 to 123 and 121 per 100 000 among those aged 80 or older (80 + ). The duration of hospitalisation due to non-invasive PnPn ranged from an average of 6.2 to 8.2 days (median 4 to 6 days, no differences between age groups). About 1 out of 10 patients were hospitalised several times for non-invasive PnPn; the proportion with re-admission ranged between 9% and 15%, depending on the age group (Table 2). There was no visual trend between the age groups and the proportion with re-admissions.

**Table 2.** The number and incidence of non-invasive pneumococcal pneumonia episodes by age group (aged 50 years and older)^a^, duration of hospitalisation (median, mean and max days), per cent re-admissions (more than 1 episodes per person), number of patients and maximum number of episodes per patient, Norway 2015–2016

Year	Age-group	Norway population size	Episodes	Number of patients	Episodes per 100 000	Days in Hospital			Readmission % (episodes/ total episodes)	Maximum number of episodes per patients
						Median	Mean	Maximum		
2015	50–59	652 318	99	96	15	5	6.8	42	12.1% (12/99)	3
2015	60–64	287 494	77	77	27	6	8.4	57	9.1% (7/77)	1
2015	65–69	277 702	121	116	44	5	7.2	37	14.0% (17/121)	3
2015	70–79	336 163	244	238	73	6	8.2	82	13.5% (33/244)	2
2015	80–89	176 933	192	190	109	6	7.6	52	10.9% (21/192)	2
2015	90 +	43 504	79	78	182	5	6.5	24	11.4% (9/79)	2
2016	50–59	661 727	103	100	16	4	6.5	38	14.6% (15/103)	3
2016	60–64	291 084	102	96	35	5	6.2	28	8.8% (9/102)	5
2016	65–69	279 227	108	108	39	5	7.3	61	5.6% (6/108)	3
2016	70–79	355 848	241	238	68	6	7.4	48	9.1% (22/241)	2
2016	80–89	176 079	213	212	121	5	7.2	50	8.0% (17/213)	2
2016	90 +	43 946	54	54	123	5	7.3	27	9.3% (5/54)	2

Episodes were based on ICD-10 discharge codes defined per person within the same 30 days.

a Data source: Norwegian Patient Register (NPR).

**Fig. 2.**
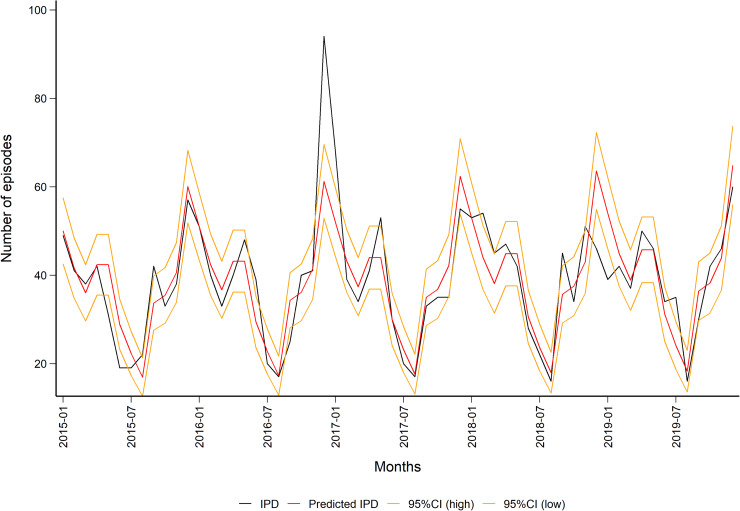
Observed (in black) and predicted (in red) monthly number of episodes of invasive pneumococcal disease in the population aged 50 years and older in Norway (2015–2019) with 95% CIs (in orange). Data source: Surveillance System for Communicable Diseases (MSIS).

We tested for an annual trend in IPD during the modelling process, but the year was not significant. The updated quasi-Poisson IPD model (1.1) predicted a seasonal trend, which is well known for the pneumococcus, with peaks during the winter months (Fig. 2, Table S2).1.1
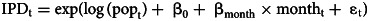

**Fig. 3.**
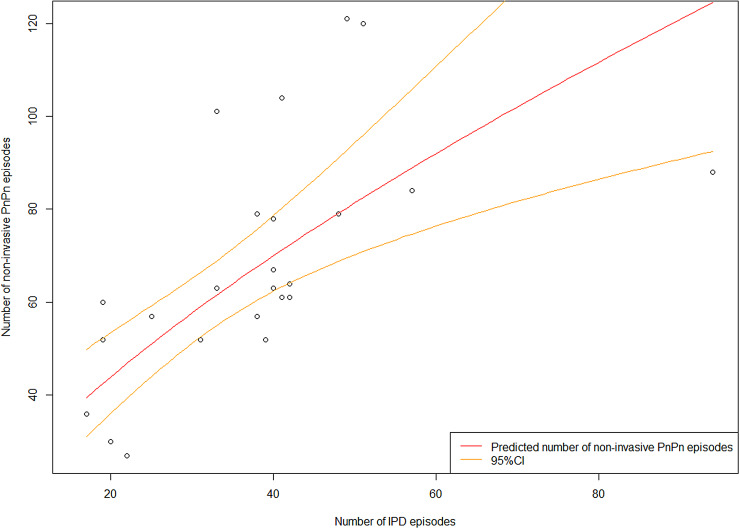
Plot of monthly number of invasive pneumococcal disease episodes in the population aged 50 years and older (50+ ) on the x-axis and non-invasive pneumococcal pneumonia episodes in the population aged 50+ on the y-axis, with the model predictions (in red) and 95% CIs (in orange) in Norway. Data source: Norwegian Patient Register (NPR; PnPn) and Surveillance System for Communicable Diseases (MSIS; IPD).

The best fitted non-invasive PnPn model was the log model (1.2). This model included intercept = 1.75 and beta1 = 0.67 (R squared = 0.50, Fig. 3).1.2
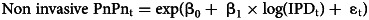

**Fig. 4.**
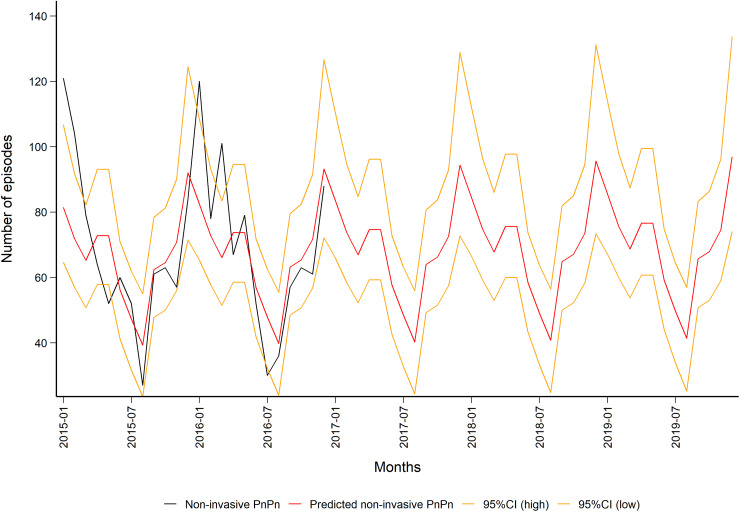
Observed (in black) and predicted (in red) monthly number of non-invasive pneumococcal pneumonia episodes in the population aged 50 years and older in Norway (2015–2019) with 95% CIs (in orange). Data source: Norwegian Patient Register (NPR).

The predicted monthly number of non-invasive PnPn ranged from a minimum of 39 (95% CI 24–55) in August to a maximum of 97 (95% CI 74–134) episodes in December, Fig. 4). Overall, we predicted 839 non-invasive PnPn cases leading to 44 episodes per 100 000 in 2019.

A sensitivity analysis was performed by excluding the numbers for December 2016, where the amount of IPD cases was especially high. This outlier coincided with a large influenza season [19]. When this outlier was excluded, year was still not significant in the IPD model (1.1), but this resulted in a better fit of the non-invasive PnPn model (1.2, *R*^2^ = 0.53), and thus a change in the range of the predicted monthly number of episodes from a minimum of 36 (95% CI 20–53) in August to a maximum of 107 (95% CI 78–156) in December, see Figs S1 and S2).

## Discussion

In this study, we used nationwide hospital discharge data for 2015–2016 to estimate simple indicators for the burden of hospitalised non-invasive PnPn among the 50+ population in Norway. The estimated annual number of non-invasive PnPn episodes per 100 000 identified in the present study is similar to those reported in other countries, with overlapping time and age groups [3, 20]. We identified that for each episode of invasive PnPn there are at least three episodes of non-invasive PnPn, which is in line with findings in other studies [5, 6]. We found that the number of episodes was 8 times higher in the oldest age group (age 80+) compared to the youngest (age 50–59), indicating that the risk of hospitalisation due to non-invasive PnPn increases with age, just like IPD [8]. This corresponds to observations reported from other countries [3, 16, 20, 21]. This higher risk with age reinforces the importance for older age groups to be vaccinated against pneumococcal disease. Persons above 65 years old (65+) are already recommended to be vaccinated, however the coverage has been quite low. Increased uptake of vaccination may prevent disease and hospitalisations in this group. Currently, people aged 50–64 years are recommended to be vaccinated only if they have comorbidities. Because of the lower incidence in this age group (age 50–65) compared to those aged 65+, a cost–effectiveness analysis would be needed to decide on a general pneumococcal vaccine recommendation.

With the many episodes of non-invasive PnPn in 2015 and 2016 (812 and 821), that on average took 6–8 days of hospitalisation, the results from this study show that the health care use due to non-invasive PnPn is substantial (*i.e.* 5000–6000 days of hospitalisation annually). Our results are comparable with the average hospital stay reported from other countries like UK and Sweden [3, 20].

The fact that non-invasive PnPn is not a notifiable disease makes it demanding to have a good overview of the episodes (cases/case definition). In the present study, NPR data for only two years were available. This is limiting possibilities for time series analysis and predictions (increases uncertainty in the models). We therefore chose to use the IPD trend to decrease uncertainty for the years we predicted, as the trend has been found to be similar for IPD and non-invasive PnPn [3, 6, 16]. The predicted monthly numbers for hospitalised non-invasive PnPn episodes 2015–2019 indicated a seasonal trend with the lowest number of episodes during summer and peaks during winter, which is well known for the pneumococcus. Our model for non-invasive PnPn underestimated the maximum and minimum monthly numbers of episodes (in comparison with the real monthly number of episodes from NPR data 2015–2016). However, our predicted number of episodes lays within the 95% CI for most months. The linear non-invasive PnPn model (1.2, Fig. 3) could explain only 50% of our data, thus the model should be interpreted with caution. Our model did not find the annual trend (year) to be significant (Fig. 2), indicating a stable number of cases over these years.

The proportion of non-invasive PnPn among hospitalised ACP estimated in the current study (5%) was very similar compared to what was found in a recent study from Sweden [20]. These authors reported that 4.7% of episodes with ACP (hospitalisation with all-cause pneumonia) were due to J13 or J18 (pneumococcal or lobar). The Swedish study used health registers and fairly similar definitions to assess the incidence of hospitalisation as in this study. Although there were some methodological differences between this and our study, the very similar numbers are indirectly confirming our results. A study evaluating administrative and clinical data of patients resident in Sicily in Italy found only 2.7% of hospitalised CAP that were reported to be attributed to PnPn, and hypothesised that that this may be due to poor sensitivity in their area, so that the real proportion is likely to be much higher [16]. Generally, it is known that the observed prevalence varies between studies conducted in different European regions [1]. Several other countries found much higher proportions of CAP being attributable to PnPn, and up to 20%–37% has been reported [1, 3, 5, 16]. There are several reasons that need to be addressed in order to understand this large gap, both biological and methodological. Pneumococcal disease is often associated with viral infections [22]. The incidence and coverage of influenza among older adults in a country may therefore affect the proportion of CAP that is attributed to pneumococci. Furthermore, the bacterial distribution causing CAP may differ along latitude. The gap between different study results may also be explained by methodological differences like the definition of CAP, coding practises and inclusion criteria for the ACP study population. Our study is based on the inclusion of ACP by ICD-codes only, while other studies also used inclusion criteria such as symptoms suggestive of lower respiratory tract infection in combination with radiography [1, 3]. Such differences may lead to a smaller denominator, and consequently a larger proportion being attributable to pneumococci. The nominator may be affected by the choice of diagnostic tests as the diagnostics for PnPn are challenging. *E.g*., a systematic review found that urine antigen tests diagnosed an additional 11% of cases beyond conventional techniques [5]. Urine antigen tests are available for clinicians in Norway, however, to which extent they are used (in hospitals), is not routinely reported. Similarly, nasopharyngeal swabs are used to support diagnostics (but sputum tests are not commonly used). However, the general susceptibility of pneumococci against antibiotics in Norway may decrease the necessity for a physician to confirm aetiology, which could lead to an underestimation of the number of PnPn diagnoses in Norway [23]. Indeed, studies with systematic testing regimes for PnPn are likely to detect more cases [3] *vs.* population-based studies based on health registers and ICD10-codes [20]. Altogether, the proportion of non-invasive PnPn among hospitalised ACP that was identified in this study is probably an underestimation, which would mean that the burden on the patient and the health care system is even higher. In addition, the burden of non-invasive PnPn is likely to increase in the near future due to a growing 50+ population in Norway as well as the rest of Europe.

The vaccine uptake in the study population (50+ ) has been low, around 15% [8]. A study conducted in Norway at the time of our study period reported that 16% of notified cases of IPD had received the PPV23 vaccine [24]. Unfortunately, we lack information on the vaccination status of the PnPn cases and are therefore unable to determine the effects of vaccination in this population. However, the incidence and number of hospital days attributed to PnPn show the need for higher pneumococcal vaccine uptake in Norway and these results will therefore be valuable for deciding on vaccine policy for the older population.

We provided simple estimates for the burden of non-invasive PnPn hospitalisation in 50+ population in Norway, thus supplementing the basis for the Norwegian guidelines for adult-vaccine policy for pneumococcal vaccination. This study indicates that the burden of non-invasive PnPn hospitalisation has a substantial impact on the health and health care use of the 50+ population in Norway, despite the childhood vaccination programme. We conclude that the recommendations and vaccine uptake for those age 65 and older should be strengthened. These results also indicate that people aged 50–64 may benefit from vaccination so it should also be considered if vaccination of adults 50–64 years would be cost-effective.

## Data Availability

The data used in this study are available on request from the corresponding author. However, because of regulations in the Norwegian Health Research Act and the Norwegian Data Protection Act for use (and storage) of Personal Data related to health, the data are not publicly available. In order to receive access to the data, the applicant will need to provide ethical approval from their IRB or equivalent body. Also, an exemption from the duty of confidentiality from Health Registry controllers in Norway is needed.

## Supplementary material

For supplementary material accompanying this paper visit http://doi.org/10.1017/S0950268822000607. click here to view supplementary material

## References

[1] Rozenbaum MH (2013) The role of *Streptococcus pneumoniae* in community-acquired pneumonia among adults in Europe: a meta-analysis. European Journal of Clinical Microbiology & Infectious Diseases 32, 305–316. 23242464 10.1007/s10096-012-1778-4

[2] Vernet G (2011) Laboratory-based diagnosis of pneumococcal pneumonia: state of the art and unmet needs. Clinical Microbiology and Infection 17(Suppl 3), 1–13. 10.1111/j.1469-0691.2011.03496.x 21457174

[3] Pick H (2020) Pneumococcal serotype trends, surveillance and risk factors in UK adult pneumonia, 2013–18. Thorax 75, 38–49. 31594801 10.1136/thoraxjnl-2019-213725

[4] The Surveillance System for Communicable Diseases (MSIS). Available at https://statistikk.fhi.no/msis (Accessed 6 September 2021).

[5] Said MA (2013) Estimating the burden of pneumococcal pneumonia among adults: a systematic review and meta-analysis of diagnostic techniques. PLoS One 8, e60273. 23565216 10.1371/journal.pone.0060273 PMC3615022

[6] van Werkhoven CH (2016) Pneumococcal conjugate vaccine herd effects on non-invasive pneumococcal pneumonia in elderly. Vaccine 34, 3275–3282. 27171754 10.1016/j.vaccine.2016.05.002

[7] Vila-Corcoles A (2020) Incidence and risk of pneumococcal pneumonia in adults with distinct underlying medical conditions: a population-based study. Lung 198, 481–489. 32253492 10.1007/s00408-020-00349-y

[8] Winje BA (2021) The risk of invasive pneumococcal disease differs between risk groups in Norway following widespread use of the 13-valent pneumococcal vaccine in children. Microorganisms 9, 1774. 34442853 10.3390/microorganisms9081774 PMC8398338

[9] Winje BA (2019) Efficacy and effectiveness of pneumococcal vaccination in elderly – an update of the literature. Report 2019. Oslo: Norwegian Institute of Public Health. Available at https://www.fhi.no/globalassets/dokumenterfiler/rapporter/2019/pneumococcal-vaccines-in-elderly-publisert.pdf (Accessed 7 September 2021).

[10] Steens A (2014) A review of the evidence to inform pneumococcal vaccine recommendations for risk groups aged 2 years and older. Epidemiology & Infection 142, 2471–2482. 24932959 10.1017/S0950268814001514 PMC9151317

[11] Aavitsland P (2019) Vaksinasjonsprogram for voksne. *Tidsskriftet Den Norske Legeforening*, Utgave 9 2019. 10.4045/tidsskr.19.0281 31140253

[12] Van Baarle D (2019) Vaccines and infectious diseases in the ageing population (VITAL) project.

[13] Méroc E (2021) European data sources for computing burden of (potential) vaccine-preventable diseases in ageing adults. BMC Infectious Diseases 21, 345. 33849461 10.1186/s12879-021-06017-7 PMC8042717

[14] Epiconcept (2021) SpIDnet project. Available at https://sites.google.com/a/epiconcept.fr/ipd-surveillance/home-2 (Accessed 30 August 2021).

[15] I-MOVE + . WP3: Pneumococcal Vaccines. Available at http://www.i-moveplus.eu/wp3 (Accessed 30 August 2021).

[16] Amodio E (2014) Estimating the burden of hospitalization for pneumococcal pneumonia in a general population aged 50 years or older and implications for vaccination strategies. Human Vaccines & Immunotherapeutics 10, 1337–1342. 24577505 10.4161/hv.27947 PMC4896611

[17] Steens A, Vestrheim DF and de Blasio BF (2015) Pneumococcal vaccination in older adults in the era of childhood vaccination: public health insights from a Norwegian statistical prediction study. Epidemics 11, 24–31. 25979279 10.1016/j.epidem.2015.01.001

[18] Steens A (2013) Prompt effect of replacing the 7-valent pneumococcal conjugate vaccine with the 13-valent vaccine on the epidemiology of invasive pneumococcal disease in Norway. Vaccine 31, 6232–6238. 24176490 10.1016/j.vaccine.2013.10.032

[19] Norwegian Institute of Public Health (2017) Influenza season in Norway 2016–17. Report 2017. Oslo: Norwegian Institute of Public Health. Available at https://www.fhi.no/globalassets/dokumenterfiler/influensa/influensa-arsrapprt-2016.pdf (Accessed 6 September 2021).

[20] Naucler P (2019) The changing epidemiology of community-acquired pneumonia: nationwide register-based study in Sweden. The Journal of Internal Medicine 286, 689–701. 31278792 10.1111/joim.12956

[21] Bechini A (2015) A retrospective analysis of hospital discharge records for *S. pneumoniae* diseases in the elderly population of florence, Italy, 2010–2012. Human Vaccines & Immunotherapeutics 11, 156–165. 25483529 10.4161/hv.34418 PMC4514361

[22] Li Y (2018) Association of seasonal viral acute respiratory infection with pneumococcal disease: a systematic review of population-based studies. BMJ Open 8, e019743. 10.1136/bmjopen-2017-019743 PMC5914779 29680810

[23] Siira L (2020) Antimicrobial susceptibility and clonality of *Streptococcus pneumoniae* isolates recovered from invasive disease cases during a period with changes in pneumococcal childhood vaccination, Norway, 2004–2016. Vaccine 38, 5454–5463. 32616324 10.1016/j.vaccine.2020.06.040

[24] Hanquet G (2019) Effect of childhood pneumococcal conjugate vaccination on invasive disease in older adults of 10 European countries: implications for adult vaccination. Thorax 74, 473–482. 30355641 10.1136/thoraxjnl-2018-211767 PMC6484683

