# Supplementary material for: Estimation of the incidence of hospitalisation for non-invasive pneumococcal pneumonia in the Norwegian population aged 50 years and older
Source: Epidemiol Infect. 2022 Apr 4;150:e81. doi: 10.1017/S0950268822000607 (PMC9044527; doi:10.1017/S0950268822000607)
Supplement: Supplementary file 1 [file S0950268822000607sup001.docx]

## Epidemiology and Infection

Estimation of the incidence of hospitalization for non-invasive pneumococcal pneumonia in the Norwegian population aged 50 years and older

Trude Marie Lyngstad*^1,2^, Anja Bråthen Kristoffersen^1^, Brita Askeland Winje^1^ and Anneke Steens^1^

^1^Norwegian Institute of Public Health, Oslo, Norway

^2^ European Programme for Intervention Epidemiology Training (EPIET), European Centre for Disease Prevention and Control (ECDC), Stockholm, Sweden

## Supplementary Material

**Table S1.** Number of invasive pneumococcal disease cases by age group (aged 50 years and older), notified to the Surveillance System for Communicable Diseases (MSIS), 2015-2019.

| **Age** | **Invasive pneumococcal disease** | | | | |
| --- | --- | --- | --- | --- | --- |
|  | **2015** | **2016** | **2017** | **2018** | **2019** |
| 50-59 | 59 | 56 | 79 | 81 | 68 |
| 60-69 | 137 | 134 | 130 | 120 | 132 |
| 70-79 | 112 | 145 | 130 | 152 | 153 |
| 80+ | 123 | 153 | 121 | 130 | 124 |
| Total for the entire population | 522 | 599 | 560 | 581 | 599 |

*Data source: Surveillance System for Communicable Diseases (MSIS)

**Table S2.** Overview of the variables, odds ratio (OR) with the 95% confidence interval (CI) and p-value obtained in the quasi-Poisson regression model for invasive pneumococcal disease (2015-2019).

| **Variables** | **OR** | **low95CI** | **high95CI** | **p-value** |
| --- | --- | --- | --- | --- |
| February | 0.83 | 0.66 | 1.03 | 0.102 |
| March | 0.72 | 0.57 | 0.90 | 0.007 |
| April | 0.84 | 0.67 | 1.05 | 0.130 |
| May | 0.84 | 0.67 | 1.05 | 0.127 |
| June | 0.57 | 0.45 | 0.73 | 0.000 |
| July | 0.44 | 0.34 | 0.58 | 0.000 |
| August | 0.33 | 0.25 | 0.45 | 0.000 |
| September | 0.66 | 0.52 | 0.84 | 0.001 |
| October | 0.70 | 0.55 | 0.88 | 0.004 |
| November | 0.80 | 0.64 | 1.00 | 0.053 |
| December | 1.18 | 0.96 | 1.44 | 0.121 |

*January is the reference months with OR = 1


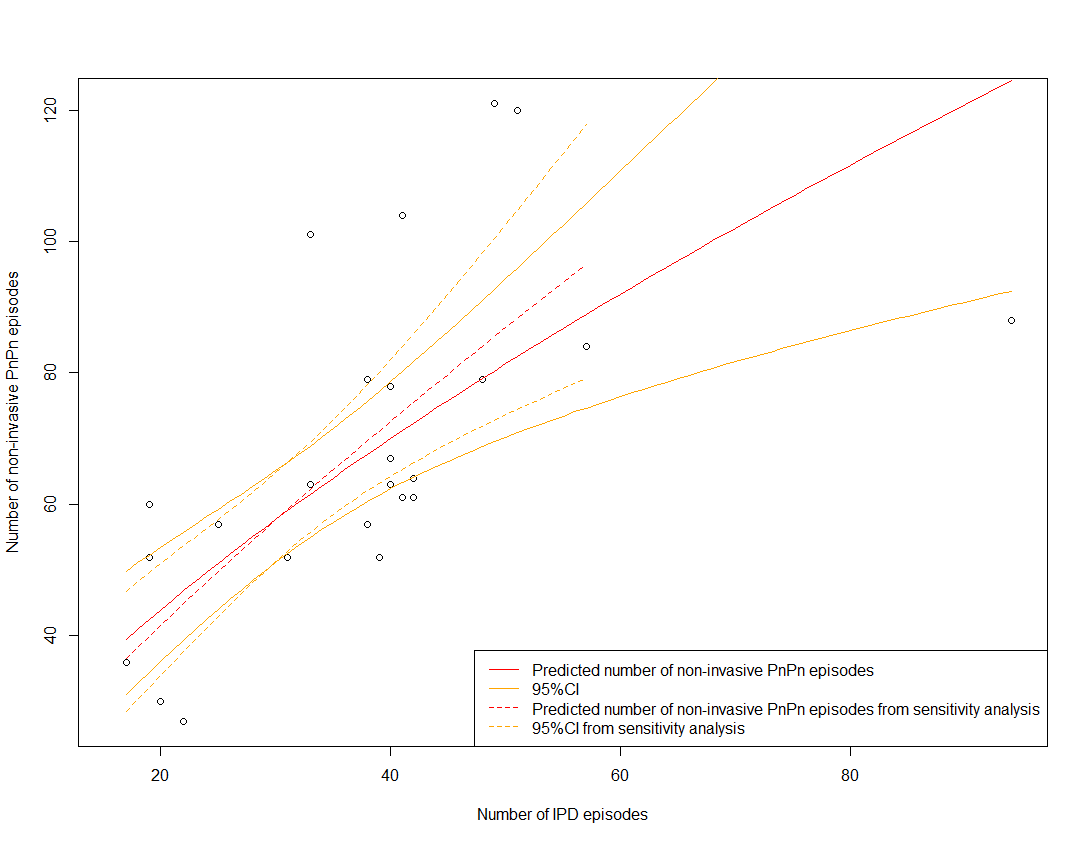


**Figure S1.** Plot of the monthly number of invasive pneumococcal disease episodes (IPD) in the population aged 50 years (50+) on the x-axis and non-invasive pneumococcal disease (PnPn) episodes in the population aged 50+ years on the y-axis (black dots) with the model predictions (in red), 95% confidence intervals (in orange) with sensitivity analysis (model run without month with maximum IPD episodes, dashed lines), in Norway. Data source: Norwegian Patient Register (NPR) and Surveillance System for Communicable Diseases (MSIS).


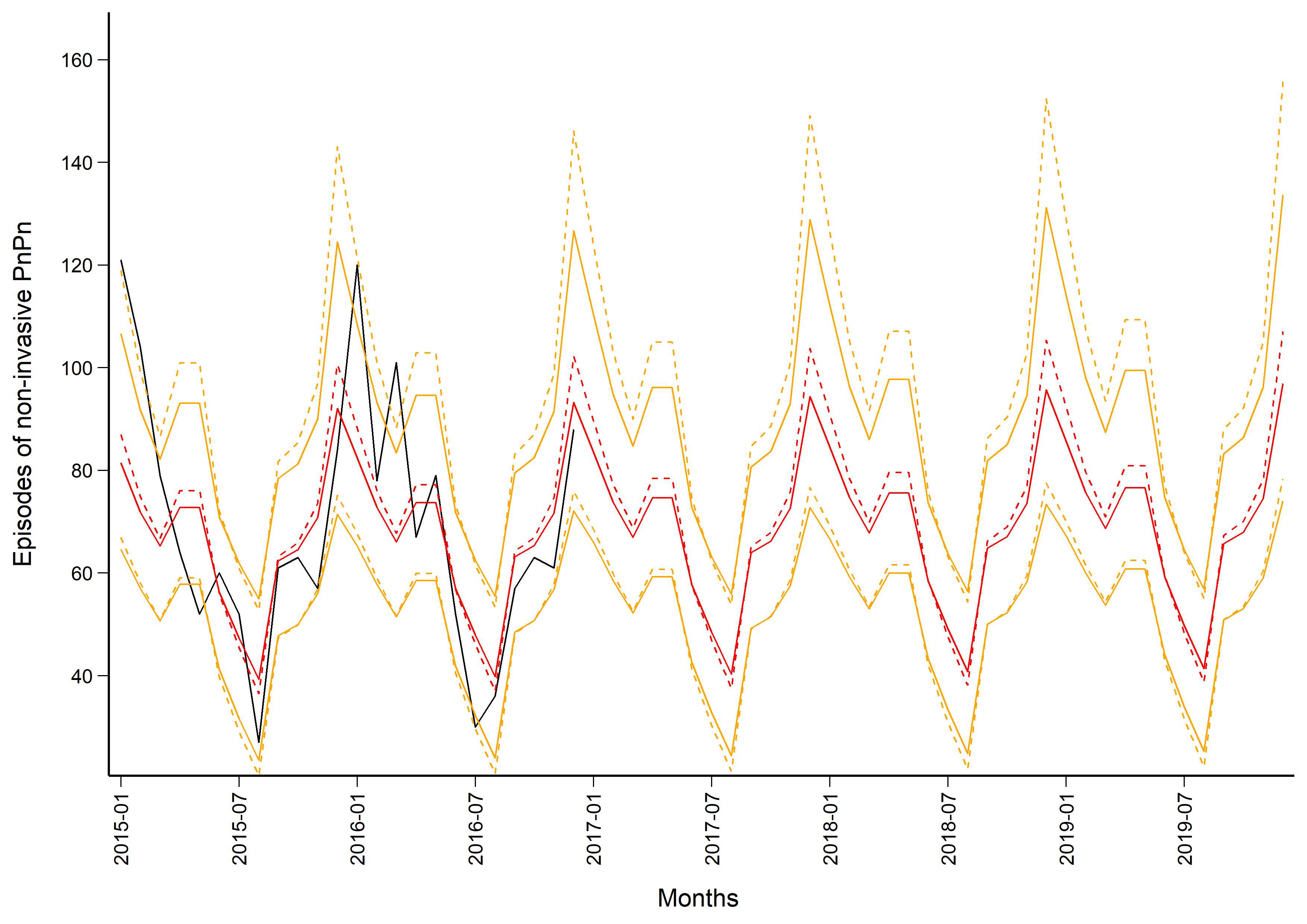


**Figure S2.** Observed (in black) and predicted (in red) monthly number of non-invasive pneumococcal pneumonia (PnPn) episodes in the population aged 50 years and older in Norway (2015 to 2019) with 95% confidence intervals (in orange) with sensitivity analysis (model run without month with maximum IPD episode, dashed lines). Data source: Norwegian Patient Register and Surveillance System for Communicable Diseases.
